# Crystal structure of 1-{3-acetyl-2-(4-chloro­phen­yl)-6-hy­droxy-4-[(2-hy­droxy­prop­yl)amino]-6-methyl­cyclo­hex-3-en-1-yl}ethanone

**DOI:** 10.1107/S2056989015008191

**Published:** 2015-04-30

**Authors:** Shaaban K. Mohamed, Joel T. Mague, Mehmet Akkurt, Antar A. Abdelhamid, Mustafa R. Albayati

**Affiliations:** a Chemistry and Environmental Division, Manchester Metropolitan University, Manchester M1 5GD, England; b Chemistry Department, Faculty of Science, Minia University, 61519 El-Minia, Egypt; c Department of Chemistry, Tulane University, New Orleans, LA 70118, USA; d Department of Physics, Faculty of Sciences, Erciyes University, 38039 Kayseri, Turkey; e Chemistry Department, Faculty of Science, Sohag University, 82524 Sohag, Egypt; f Kirkuk University, College of Science, Department of Chemistry, Kirkuk, Iraq

**Keywords:** crystal structure, 1,3-diketones, hydrogen bonding

## Abstract

In the title compound, C_20_H_26_ClNO_4_, the central cyclo­hexene ring adopts an approximate envelope conformation with the C atom binding with the hy­droxy group at the tip of the flap. There is an intramolecular N—H⋯O hydrogen bond generating an *S*(6) ring motif. In the crystal, classical O—H⋯O hydrogen bonds and weak C—H⋯O and C—H⋯Cl inter­actions link the mol­ecules, forming a three-dimensional supra­molecular architecture. The crystal structure was refined as a four-component twin.

## Related literature   

For use of 1,3-diketones as building block in mutasynthesis and as chelating ligands, see: Bergé *et al.* (1997 ▸); Nagpal *et al.* (2001 ▸); Simoni *et al.* (1999 ▸); Garnovskii *et al.* (1999 ▸).
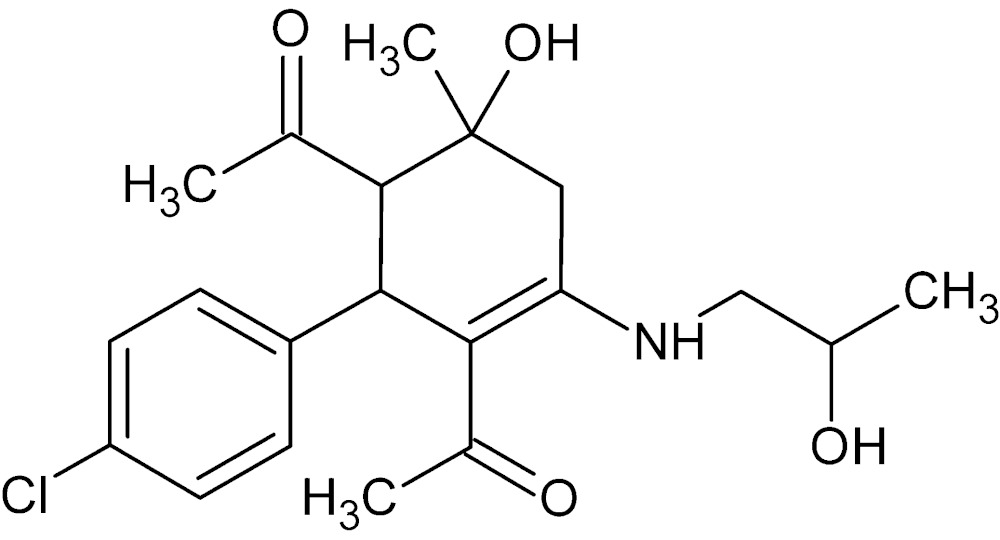


## Experimental   

### Crystal data  

C_20_H_26_ClNO_4_
*M* *_r_* = 379.87
Monoclinic, 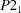

*a* = 5.5490 (2) Å
*b* = 8.7759 (3) Å
*c* = 19.4428 (6) Å
β = 92.815 (2)°
*V* = 945.67 (6) Å^3^
*Z* = 2
Cu *K*α radiation
μ = 1.98 mm^−1^
*T* = 150 K
0.26 × 0.18 × 0.02 mm

### Data collection  

Bruker D8 VENTURE PHOTON 100 CMOS diffractometer
Absorption correction: multi-scan (*SADABS*; Bruker, 2014 ▸) *T*_min_ = 0.63, *T*_max_ = 0.97
7079 measured reflections
7079 independent reflections
6072 reflections with *I* > 2σ(*I*)

### Refinement  

*R*[*F* ^2^ > 2σ(*F* ^2^)] = 0.059
*wR*(*F* ^2^) = 0.144
*S* = 1.06
7079 reflections
242 parameters
1 restraint
H-atom parameters constrained
Δρ_max_ = 0.39 e Å^−3^
Δρ_min_ = −0.38 e Å^−3^
Absolute structure: The crystal is a non-merohedral twin with each component being a racemic twin as well.
Absolute structure parameter: 0.033 (15)

### 

Data collection: *APEX2* (Bruker, 2014 ▸); cell refinement: *SAINT* (Bruker, 2014 ▸); data reduction: *SAINT*; program(s) used to solve structure: *SHELXT* (Sheldrick, 2015*a* ▸); program(s) used to refine structure: *SHELXL2014* (Sheldrick, 2015*b* ▸); molecular graphics: *DIAMOND* (Brandenburg & Putz, 2012 ▸); software used to prepare material for publication: *SHELXTL* (Sheldrick, 2008 ▸).

## Supplementary Material

Crystal structure: contains datablock(s) global, I. DOI: 10.1107/S2056989015008191/xu5848sup1.cif

Structure factors: contains datablock(s) I. DOI: 10.1107/S2056989015008191/xu5848Isup2.hkl

Supporting information file. DOI: 10.1107/S2056989015008191/xu5848Isup3.cml

**Fig. 1.**
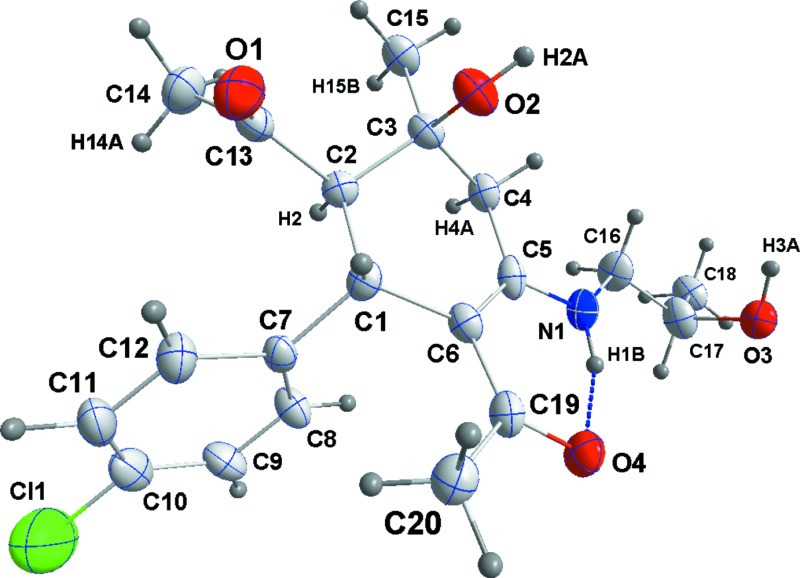
The title molecule with labeling scheme and 50% probability ellipsoids. The intramolecular N—H···O hydrogen bond is shown as a dotted line.

**Fig. 2.**
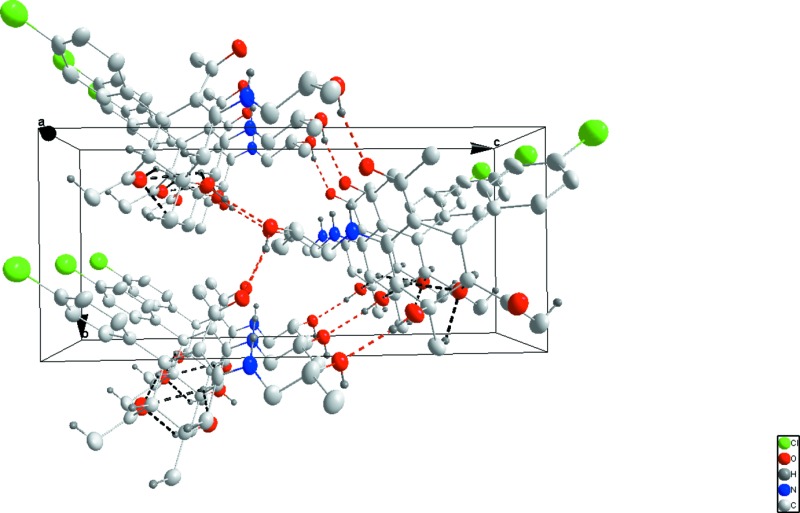
Packing viewed down the a axis. O—H···O hydrogen bonds are shown as red dotted lines.

. DOI: 10.1107/S2056989015008191/xu5848fig1.tif
The title mol­ecule with labeling scheme and 50% probability ellipsoids. The intra­molecular N—H⋯O hydrogen bond is shown as a dotted line.

a . DOI: 10.1107/S2056989015008191/xu5848fig2.tif
Packing viewed down the *a* axis. O—H⋯O hydrogen bonds are shown as red dotted lines.

**Fig. 3.**
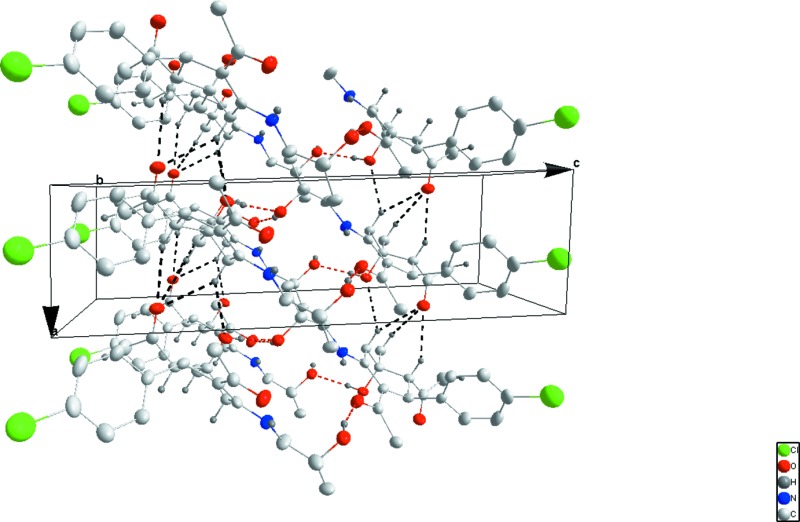
Packing showing the "three-point" C—H···O interactions as black dotted lines.

. DOI: 10.1107/S2056989015008191/xu5848fig3.tif
Packing showing the "three-point" C—H⋯O inter­actions as black dotted lines.

CCDC reference: 1061756

Additional supporting information:  crystallographic information; 3D view; checkCIF report

## Figures and Tables

**Table 1:** Hydrogen-bond geometry (, )

*D*H*A*	*D*H	H*A*	*D* *A*	*D*H*A*
O2H2*A*O3^i^	0.84	1.97	2.811(6)	174
O3H3*A*O4^i^	0.84	1.95	2.768(6)	164
N1H1*B*O4	0.91	1.82	2.601(7)	142
C2H2O1^ii^	1.00	2.61	3.488(6)	147
C4H4*A*O1^ii^	0.99	2.58	3.456(7)	147
C4H4*A*O2^ii^	0.99	2.57	3.347(6)	136
C14H14*A*Cl1^iii^	0.98	2.98	3.818(7)	145
C15H15*B*O1^ii^	0.98	2.62	3.473(8)	146

Symmetry codes: (i) 
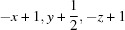
; (ii) 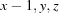; (iii) 
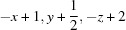
.

## supplementary crystallographic information

### S1. Comment

1,3-Diketones are important building block, and their usefulness in cyclic and heterocyclic preparations has been largely illustrated (Bergé *et al.*, 1997; Nagpal *et al.*, 2001; Simoni *et al.*, 1999). Also, 1,3-diketones are key structural units in many chelating ligand for lanthanide and transition metals (Garnovskii *et al.*, 1999). In this concept, we report in this study the synthesis and crystal structure study of the title compound.

In the title molecule, the central six-membered ring adopts an approximate envelope conformation with C3 at the tip of the "flap" (Fig. 1). A Cremer-Pople puckering analysis gives a puckering amplitude Q = 0.530 (6) Å and additional parameters θ = 58.7 (6)° and φ = 109.3 (8)°. The conformation of the 2-hydroxypropylamino side chain is determined in part by the intramolecular N1—H1B···O4 hydrogen bonds. Two intermolecular hydrogen bonds (O2—H2A···O3^i^ and O3—H3A···O4^i^ (i: 1 - *x*, 1/2 + *y*, 1 - *z*) form a unit which is propagated by the 2_1_ axis using further pairs of these hydrogen bonds to generate layers approximately parallel to (101) (Fig. 2). In addition, there are intermolecular "three point" C—H···O interactions between the central cyclohexene ring and O1 parallel to the *a* axis (Fig. 3).

### S2. Experimental

A mixture of 4-chlorobenzaldehyde (1 mmol, 140 mg), 1-aminopropan-2-ol (1 mmol, 75 mg) and pentane-2,4-dione (1 mmol, 100 mg) in 30 ml e thanol was refluxed for 2 h. The resulting solid product was collected, dried under vacuum and recrystallized from ethanol to afford colourless crystals in excelent yield (92%).

### S3. Refinement

H-atoms attached to carbon were placed in calculated positions (C—H = 0.95 - 0.98 Å) while those attached to nitrogen and oxygen were placed in locations derived from a difference map and their parameters adjusted to give N—H = 0.91 and O—H = 0.84 Å. All were included as riding contributions with isotropic displacement parameters 1.2 - 1.5 times those of the attached atoms. In the final stages of the refinement it became evident that not only was the crystal twinned by a 180° rotation about the *c** axis but also each of these components was a racemic twin. Consequently, the model was finally refined as a 4-component twin.

### Crystal data

**Table:**

C_20_H_26_ClNO_4_	*F*(000) = 404
*M**_r_* = 379.87	*D*_x_ = 1.323 Mg m^−^^3^
Monoclinic, *P*2_1_	Cu *K*α radiation, λ = 1.54178 Å
*a* = 5.5490 (2) Å	Cell parameters from 5259 reflections
*b* = 8.7759 (3) Å	θ = 4.6–72.4°
*c* = 19.4428 (6) Å	µ = 1.98 mm^−^^1^
β = 92.815 (2)°	*T* = 150 K
*V* = 945.67 (6) Å^3^	Plate, colourless
*Z* = 2	0.26 × 0.18 × 0.02 mm

### Data collection

**Table:**

Bruker D8 VENTURE PHOTON 100 CMOS diffractometer	7079 measured reflections
Radiation source: INCOATEC IµS micro–focus source	7079 independent reflections
Mirror monochromator	6072 reflections with *I* > 2σ(*I*)
Detector resolution: 10.4167 pixels mm^-1^	θ_max_ = 72.5°, θ_min_ = 4.6°
ω scans	*h* = −6→6
Absorption correction: multi-scan (*SADABS*; Bruker, 2014)	*k* = −10→10
*T*_min_ = 0.63, *T*_max_ = 0.97	*l* = −24→24

### Refinement

**Table:**

Refinement on *F*^2^	Secondary atom site location: difference Fourier map
Least-squares matrix: full	Hydrogen site location: inferred from neighbouring sites
*R*[*F*^2^ > 2σ(*F*^2^)] = 0.059	H-atom parameters constrained
*wR*(*F*^2^) = 0.144	*w* = 1/[σ^2^(*F*_o_^2^) + (0.0693*P*)^2^ + 0.3038*P*] where *P* = (*F*_o_^2^ + 2*F*_c_^2^)/3
*S* = 1.06	(Δ/σ)_max_ = 0.001
7079 reflections	Δρ_max_ = 0.39 e Å^−^^3^
242 parameters	Δρ_min_ = −0.38 e Å^−^^3^
1 restraint	Absolute structure: The crystal is a non-merohedral twin with each component being a racemic twin as well.
Primary atom site location: structure-invariant direct methods	Absolute structure parameter: 0.033 (15)

### Special details

**Table:**

Experimental. Analysis of 1837 reflections having I/σ(I) > 12 and chosen from the full data set with *CELL_NOW* (Sheldrick, 2008) showed the crystal to belong to the monoclinic system and to be twinned by a 180° rotation about the *c** axis. The raw data were processed using the multi-component version of *SAINT* under control of the two-component orientation file generated by *CELL_NOW*.
Geometry. All e.s.d.'s (except the e.s.d. in the dihedral angle between two l.s. planes) are estimated using the full covariance matrix. The cell e.s.d.'s are taken into account individually in the estimation of e.s.d.'s in distances, angles and torsion angles; correlations between e.s.d.'s in cell parameters are only used when they are defined by crystal symmetry. An approximate (isotropic) treatment of cell e.s.d.'s is used for estimating e.s.d.'s involving l.s. planes.
Refinement. Refinement of *F*^2^ against ALL reflections. The weighted *R*-factor *wR* and goodness of fit *S* are based on *F*^2^, conventional *R*-factors *R* are based on *F*, with *F* set to zero for negative *F*^2^. The threshold expression of *F*^2^ > σ(*F*^2^) is used only for calculating *R*-factors(gt) *etc*. and is not relevant to the choice of reflections for refinement. *R*-factors based on *F*^2^ are statistically about twice as large as those based on *F*, and *R*- factors based on ALL data will be even larger. H-atoms attached to carbon were placed in calculated positions (C—H = 0.95 - 0.98 Å) while those attached to oxygen were placed in locations derived from a difference map and their parameters adjusted to give O—H = 0.84 Å. All were included as riding contributions with isotropic displacement parameters 1.2 - 1.5 times those of the attached atoms. In the final stages of the refinement it became evident that not only was the crystal twinned by a 180° rotation about the *c** axis but also each of these components was a racemic twin. Consequently, the model was finally refined as a 4-component twin.

### Fractional atomic coordinates and isotropic or equivalent isotropic displacement parameters (Å^2^)

**Table:**

	*x*	*y*	*z*	*U*_iso_*/*U*_eq_	
Cl1	0.6307 (4)	0.0955 (2)	1.00356 (9)	0.0579 (6)	
O1	1.1051 (7)	0.7167 (5)	0.8132 (2)	0.0376 (11)	
O2	0.8526 (6)	0.7591 (5)	0.6729 (2)	0.0320 (10)	
H2A	0.8220	0.8176	0.6394	0.038*	
O3	0.2467 (7)	0.4376 (5)	0.4451 (2)	0.0332 (10)	
H3A	0.2914	0.5269	0.4366	0.040*	
O4	0.6555 (7)	0.2231 (5)	0.6072 (2)	0.0341 (10)	
N1	0.3747 (8)	0.4595 (7)	0.5929 (2)	0.0312 (12)	
H1B	0.4164	0.3602	0.5871	0.037*	
C1	0.7897 (9)	0.4895 (7)	0.7530 (3)	0.0248 (12)	
H1	0.9672	0.4963	0.7468	0.030*	
C2	0.7024 (9)	0.6487 (6)	0.7741 (3)	0.0251 (12)	
H2	0.5523	0.6351	0.7998	0.030*	
C3	0.6400 (9)	0.7509 (7)	0.7111 (3)	0.0264 (13)	
C4	0.4393 (9)	0.6728 (7)	0.6691 (3)	0.0282 (13)	
H4A	0.2917	0.6734	0.6957	0.034*	
H4B	0.4045	0.7322	0.6265	0.034*	
C5	0.4944 (10)	0.5119 (8)	0.6498 (3)	0.0288 (14)	
C6	0.6716 (9)	0.4266 (7)	0.6858 (3)	0.0256 (12)	
C7	0.7523 (10)	0.3846 (7)	0.8148 (3)	0.0255 (12)	
C8	0.5421 (11)	0.3006 (8)	0.8207 (3)	0.0337 (14)	
H8	0.4212	0.3047	0.7845	0.040*	
C9	0.5031 (12)	0.2112 (8)	0.8778 (3)	0.0385 (15)	
H9	0.3601	0.1523	0.8802	0.046*	
C10	0.6767 (12)	0.2094 (8)	0.9311 (3)	0.0390 (16)	
C11	0.8864 (12)	0.2898 (8)	0.9274 (3)	0.0407 (17)	
H11	1.0056	0.2856	0.9641	0.049*	
C12	0.9244 (11)	0.3778 (8)	0.8695 (3)	0.0361 (15)	
H12	1.0699	0.4341	0.8670	0.043*	
C13	0.8910 (10)	0.7249 (7)	0.8224 (3)	0.0292 (13)	
C14	0.8022 (13)	0.8077 (9)	0.8835 (3)	0.0424 (17)	
H14A	0.7646	0.7340	0.9193	0.064*	
H14B	0.6566	0.8654	0.8698	0.064*	
H14C	0.9274	0.8778	0.9016	0.064*	
C15	0.5570 (10)	0.9097 (8)	0.7316 (3)	0.0342 (14)	
H15A	0.6861	0.9602	0.7592	0.051*	
H15B	0.4131	0.9011	0.7586	0.051*	
H15C	0.5183	0.9698	0.6900	0.051*	
C16	0.1783 (11)	0.5437 (8)	0.5557 (3)	0.0362 (15)	
H16A	0.2408	0.6421	0.5392	0.043*	
H16B	0.0486	0.5657	0.5875	0.043*	
C17	0.0785 (11)	0.4540 (9)	0.4965 (3)	0.0403 (17)	
H17	0.0439	0.3496	0.5140	0.048*	
C18	−0.1597 (10)	0.5198 (8)	0.4682 (3)	0.0367 (15)	
H18A	−0.1334	0.6231	0.4510	0.055*	
H18B	−0.2745	0.5234	0.5049	0.055*	
H18C	−0.2249	0.4554	0.4305	0.055*	
C19	0.7529 (10)	0.2866 (7)	0.6595 (3)	0.0278 (13)	
C20	0.9775 (10)	0.2105 (8)	0.6928 (3)	0.0302 (13)	
H20A	0.9942	0.1077	0.6739	0.045*	
H20B	0.9617	0.2042	0.7427	0.045*	
H20C	1.1205	0.2709	0.6831	0.045*	

### Atomic displacement parameters (Å^2^)

**Table:**

	*U*^11^	*U*^22^	*U*^33^	*U*^12^	*U*^13^	*U*^23^
Cl1	0.0853 (14)	0.0563 (11)	0.0340 (8)	0.0254 (10)	0.0201 (8)	0.0185 (9)
O1	0.025 (2)	0.048 (3)	0.039 (2)	−0.007 (2)	−0.0011 (17)	−0.002 (2)
O2	0.0218 (19)	0.039 (3)	0.035 (2)	0.0045 (18)	0.0055 (16)	0.009 (2)
O3	0.028 (2)	0.040 (3)	0.032 (2)	0.001 (2)	−0.0011 (16)	−0.002 (2)
O4	0.034 (2)	0.039 (3)	0.029 (2)	0.003 (2)	−0.0077 (16)	−0.004 (2)
N1	0.023 (2)	0.044 (3)	0.026 (2)	0.006 (2)	−0.0041 (18)	−0.001 (2)
C1	0.018 (2)	0.030 (3)	0.026 (3)	0.000 (2)	−0.002 (2)	−0.003 (2)
C2	0.018 (2)	0.030 (3)	0.027 (3)	0.000 (2)	0.003 (2)	0.001 (3)
C3	0.020 (3)	0.031 (3)	0.029 (3)	0.001 (2)	0.003 (2)	0.005 (3)
C4	0.019 (3)	0.038 (4)	0.027 (3)	0.002 (2)	0.000 (2)	0.004 (3)
C5	0.020 (3)	0.047 (4)	0.020 (3)	0.002 (3)	0.004 (2)	0.002 (3)
C6	0.020 (2)	0.032 (3)	0.025 (3)	0.001 (2)	−0.001 (2)	0.005 (3)
C7	0.026 (3)	0.027 (3)	0.023 (3)	0.008 (2)	−0.002 (2)	0.000 (2)
C8	0.028 (3)	0.040 (4)	0.032 (3)	0.003 (3)	−0.001 (2)	0.011 (3)
C9	0.038 (3)	0.036 (4)	0.042 (4)	0.007 (3)	0.007 (3)	0.013 (3)
C10	0.049 (4)	0.041 (4)	0.028 (3)	0.014 (3)	0.010 (3)	0.002 (3)
C11	0.052 (4)	0.041 (4)	0.028 (3)	0.012 (3)	−0.011 (3)	0.001 (3)
C12	0.036 (3)	0.043 (4)	0.029 (3)	−0.001 (3)	−0.007 (2)	0.000 (3)
C13	0.029 (3)	0.030 (3)	0.028 (3)	−0.005 (3)	−0.001 (2)	0.006 (3)
C14	0.048 (4)	0.045 (4)	0.034 (3)	−0.001 (3)	0.002 (3)	−0.009 (3)
C15	0.029 (3)	0.038 (4)	0.036 (3)	0.007 (3)	0.003 (2)	0.007 (3)
C16	0.027 (3)	0.046 (4)	0.035 (3)	0.011 (3)	−0.008 (2)	−0.001 (3)
C17	0.029 (3)	0.057 (5)	0.034 (3)	0.006 (3)	0.000 (3)	0.008 (3)
C18	0.022 (3)	0.049 (4)	0.039 (4)	−0.002 (3)	−0.003 (2)	0.008 (3)
C19	0.025 (3)	0.036 (3)	0.022 (3)	0.000 (3)	0.001 (2)	0.001 (3)
C20	0.025 (3)	0.035 (3)	0.030 (3)	0.003 (3)	0.001 (2)	−0.003 (3)

### Geometric parameters (Å, º)

**Table:**

Cl1—C10	1.756 (7)	C8—H8	0.9500
O1—C13	1.212 (7)	C9—C10	1.381 (9)
O2—C3	1.427 (6)	C9—H9	0.9500
O2—H2A	0.8400	C10—C11	1.366 (10)
O3—C17	1.407 (7)	C11—C12	1.390 (9)
O3—H3A	0.8404	C11—H11	0.9500
O4—C19	1.257 (7)	C12—H12	0.9500
N1—C5	1.342 (7)	C13—C14	1.497 (9)
N1—C16	1.477 (7)	C14—H14A	0.9800
N1—H1B	0.9100	C14—H14B	0.9800
C1—C6	1.534 (7)	C14—H14C	0.9800
C1—C7	1.537 (8)	C15—H15A	0.9800
C1—C2	1.541 (8)	C15—H15B	0.9800
C1—H1	1.0000	C15—H15C	0.9800
C2—C13	1.526 (7)	C16—C17	1.480 (9)
C2—C3	1.543 (8)	C16—H16A	0.9900
C2—H2	1.0000	C16—H16B	0.9900
C3—C4	1.513 (8)	C17—C18	1.521 (8)
C3—C15	1.526 (9)	C17—H17	1.0000
C4—C5	1.497 (9)	C18—H18A	0.9800
C4—H4A	0.9900	C18—H18B	0.9800
C4—H4B	0.9900	C18—H18C	0.9800
C5—C6	1.396 (8)	C19—C20	1.530 (8)
C6—C19	1.414 (9)	C20—H20A	0.9800
C7—C8	1.389 (8)	C20—H20B	0.9800
C7—C12	1.394 (8)	C20—H20C	0.9800
C8—C9	1.385 (9)		
			
C3—O2—H2A	107.1	C10—C11—H11	120.2
C17—O3—H3A	104.9	C12—C11—H11	120.2
C5—N1—C16	123.8 (6)	C11—C12—C7	121.0 (6)
C5—N1—H1B	108.2	C11—C12—H12	119.5
C16—N1—H1B	127.2	C7—C12—H12	119.5
C6—C1—C7	112.5 (5)	O1—C13—C14	120.7 (5)
C6—C1—C2	115.2 (5)	O1—C13—C2	122.0 (5)
C7—C1—C2	106.1 (4)	C14—C13—C2	117.3 (5)
C6—C1—H1	107.6	C13—C14—H14A	109.5
C7—C1—H1	107.6	C13—C14—H14B	109.5
C2—C1—H1	107.6	H14A—C14—H14B	109.5
C13—C2—C1	110.3 (4)	C13—C14—H14C	109.5
C13—C2—C3	110.7 (5)	H14A—C14—H14C	109.5
C1—C2—C3	112.2 (5)	H14B—C14—H14C	109.5
C13—C2—H2	107.8	C3—C15—H15A	109.5
C1—C2—H2	107.8	C3—C15—H15B	109.5
C3—C2—H2	107.8	H15A—C15—H15B	109.5
O2—C3—C4	110.3 (5)	C3—C15—H15C	109.5
O2—C3—C15	111.0 (5)	H15A—C15—H15C	109.5
C4—C3—C15	109.3 (5)	H15B—C15—H15C	109.5
O2—C3—C2	106.4 (4)	N1—C16—C17	110.7 (5)
C4—C3—C2	107.2 (5)	N1—C16—H16A	109.5
C15—C3—C2	112.6 (5)	C17—C16—H16A	109.5
C5—C4—C3	114.2 (5)	N1—C16—H16B	109.5
C5—C4—H4A	108.7	C17—C16—H16B	109.5
C3—C4—H4A	108.7	H16A—C16—H16B	108.1
C5—C4—H4B	108.7	O3—C17—C16	111.8 (5)
C3—C4—H4B	108.7	O3—C17—C18	112.2 (5)
H4A—C4—H4B	107.6	C16—C17—C18	111.4 (6)
N1—C5—C6	122.5 (6)	O3—C17—H17	107.0
N1—C5—C4	115.5 (5)	C16—C17—H17	107.0
C6—C5—C4	121.8 (5)	C18—C17—H17	107.0
C5—C6—C19	120.8 (5)	C17—C18—H18A	109.5
C5—C6—C1	119.7 (6)	C17—C18—H18B	109.5
C19—C6—C1	119.4 (5)	H18A—C18—H18B	109.5
C8—C7—C12	117.4 (6)	C17—C18—H18C	109.5
C8—C7—C1	121.9 (5)	H18A—C18—H18C	109.5
C12—C7—C1	120.5 (5)	H18B—C18—H18C	109.5
C9—C8—C7	122.1 (6)	O4—C19—C6	123.1 (5)
C9—C8—H8	118.9	O4—C19—C20	117.2 (5)
C7—C8—H8	118.9	C6—C19—C20	119.6 (5)
C10—C9—C8	118.5 (6)	C19—C20—H20A	109.5
C10—C9—H9	120.7	C19—C20—H20B	109.5
C8—C9—H9	120.7	H20A—C20—H20B	109.5
C11—C10—C9	121.2 (6)	C19—C20—H20C	109.5
C11—C10—Cl1	119.7 (5)	H20A—C20—H20C	109.5
C9—C10—Cl1	119.0 (5)	H20B—C20—H20C	109.5
C10—C11—C12	119.6 (6)		
			
C6—C1—C2—C13	−157.7 (5)	C6—C1—C7—C8	−34.3 (7)
C7—C1—C2—C13	77.1 (5)	C2—C1—C7—C8	92.5 (6)
C6—C1—C2—C3	−33.8 (6)	C6—C1—C7—C12	149.6 (5)
C7—C1—C2—C3	−159.0 (4)	C2—C1—C7—C12	−83.6 (6)
C13—C2—C3—O2	66.3 (6)	C12—C7—C8—C9	−0.9 (9)
C1—C2—C3—O2	−57.4 (6)	C1—C7—C8—C9	−177.1 (6)
C13—C2—C3—C4	−175.7 (4)	C7—C8—C9—C10	1.8 (10)
C1—C2—C3—C4	60.7 (6)	C8—C9—C10—C11	−2.1 (10)
C13—C2—C3—C15	−55.5 (6)	C8—C9—C10—Cl1	−179.6 (5)
C1—C2—C3—C15	−179.1 (5)	C9—C10—C11—C12	1.4 (10)
O2—C3—C4—C5	61.4 (6)	Cl1—C10—C11—C12	178.9 (5)
C15—C3—C4—C5	−176.4 (5)	C10—C11—C12—C7	−0.4 (10)
C2—C3—C4—C5	−54.1 (6)	C8—C7—C12—C11	0.1 (9)
C16—N1—C5—C6	176.8 (6)	C1—C7—C12—C11	176.5 (6)
C16—N1—C5—C4	−7.7 (8)	C1—C2—C13—O1	41.0 (7)
C3—C4—C5—N1	−154.8 (5)	C3—C2—C13—O1	−83.7 (7)
C3—C4—C5—C6	20.7 (7)	C1—C2—C13—C14	−137.3 (6)
N1—C5—C6—C19	7.2 (9)	C3—C2—C13—C14	98.0 (6)
C4—C5—C6—C19	−168.0 (5)	C5—N1—C16—C17	−178.4 (6)
N1—C5—C6—C1	−176.1 (5)	N1—C16—C17—O3	−67.3 (7)
C4—C5—C6—C1	8.7 (8)	N1—C16—C17—C18	166.3 (5)
C7—C1—C6—C5	120.3 (6)	C5—C6—C19—O4	−7.9 (9)
C2—C1—C6—C5	−1.5 (8)	C1—C6—C19—O4	175.4 (5)
C7—C1—C6—C19	−63.0 (7)	C5—C6—C19—C20	168.7 (5)
C2—C1—C6—C19	175.2 (5)	C1—C6—C19—C20	−7.9 (8)

### Hydrogen-bond geometry (Å, º)

**Table:**

*D*—H···*A*	*D*—H	H···*A*	*D*···*A*	*D*—H···*A*
O2—H2*A*···O3^i^	0.84	1.97	2.811 (6)	174
O3—H3*A*···O4^i^	0.84	1.95	2.768 (6)	164
N1—H1*B*···O4	0.91	1.82	2.601 (7)	142
C2—H2···O1^ii^	1.00	2.61	3.488 (6)	147
C4—H4*A*···O1^ii^	0.99	2.58	3.456 (7)	147
C4—H4*A*···O2^ii^	0.99	2.57	3.347 (6)	136
C14—H14*A*···Cl1^iii^	0.98	2.98	3.818 (7)	145
C15—H15*B*···O1^ii^	0.98	2.62	3.473 (8)	146

Symmetry codes: (i) −*x*+1, *y*+1/2, −*z*+1; (ii) *x*−1, *y*, *z*; (iii) −*x*+1, *y*+1/2, −*z*+2.
