# Supplementary figures and images for: Crystal structure of 1-{3-acetyl-2-(4-chloro­phen­yl)-6-hy­droxy-4-[(2-hy­droxy­prop­yl)amino]-6-methyl­cyclo­hex-3-en-1-yl}ethanone
Source: Acta Crystallogr E Crystallogr Commun. 2015 Apr 30;71(Pt 5):o369–70. doi: 10.1107/S2056989015008191 (PMC4420087; doi:10.1107/S2056989015008191)

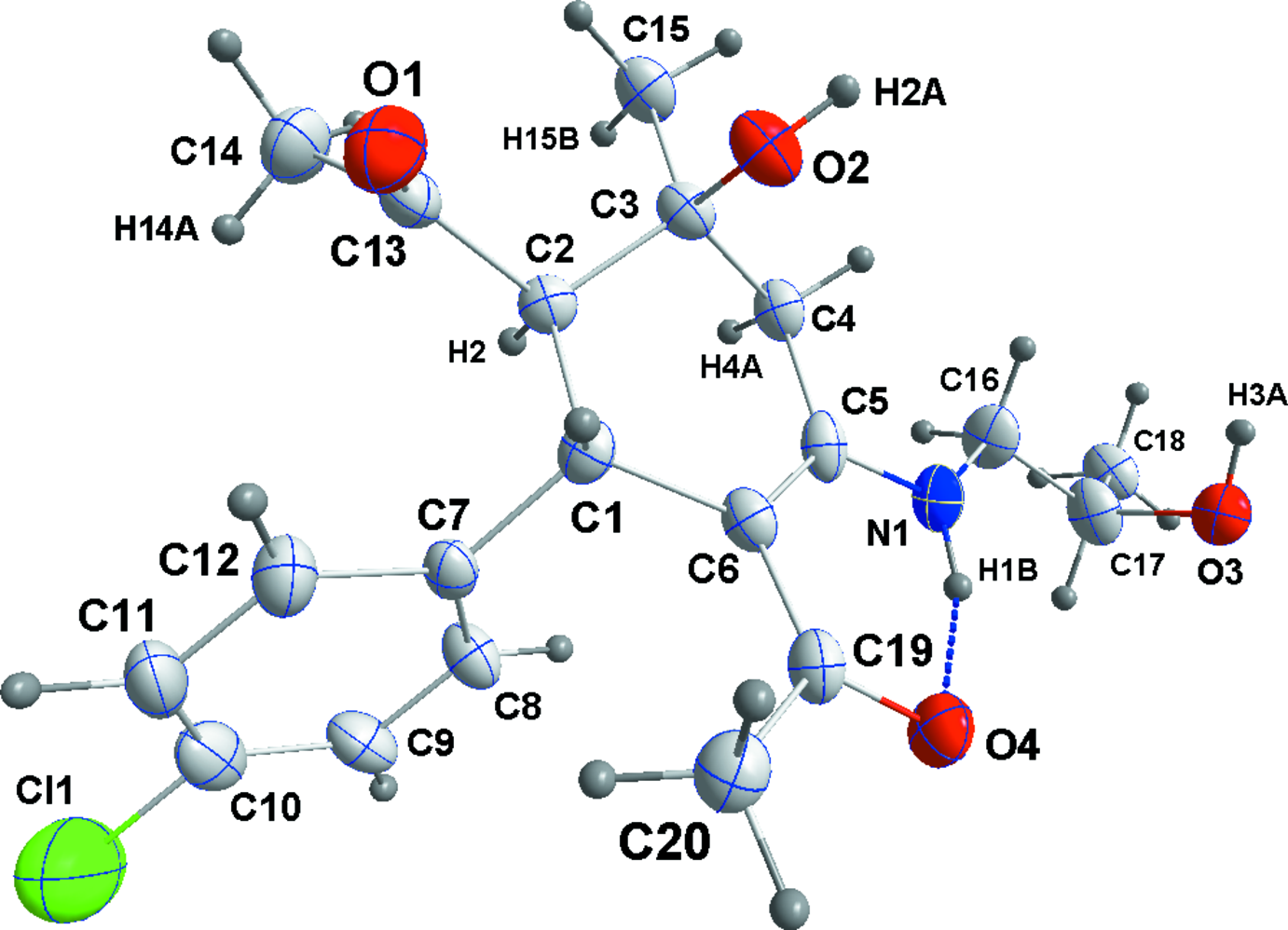

Supplement: Supplementary file 4 [file e-71-0o369-fig1.tif]

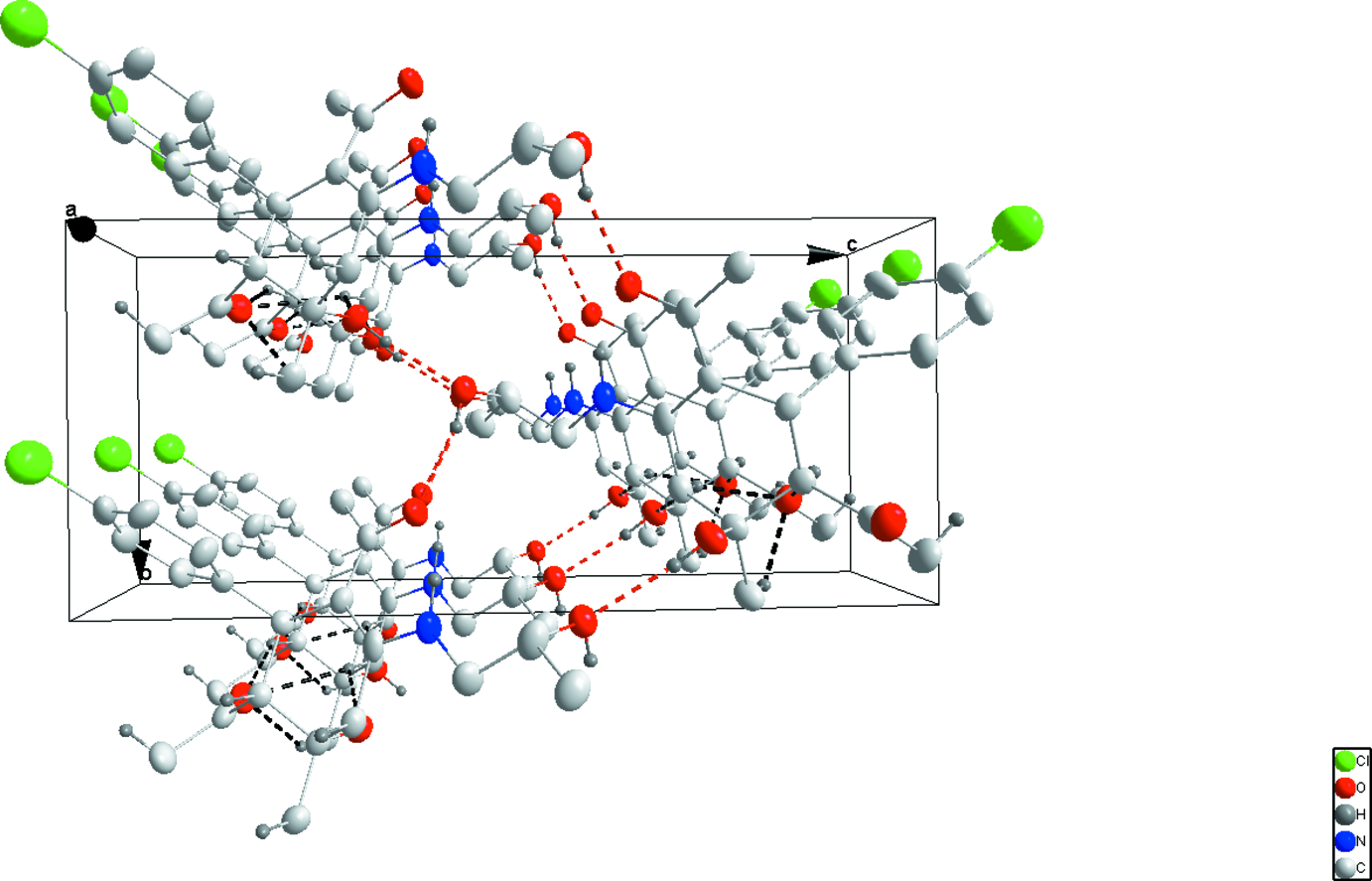

Supplement: Supplementary file 5 [file e-71-0o369-fig2.tif]

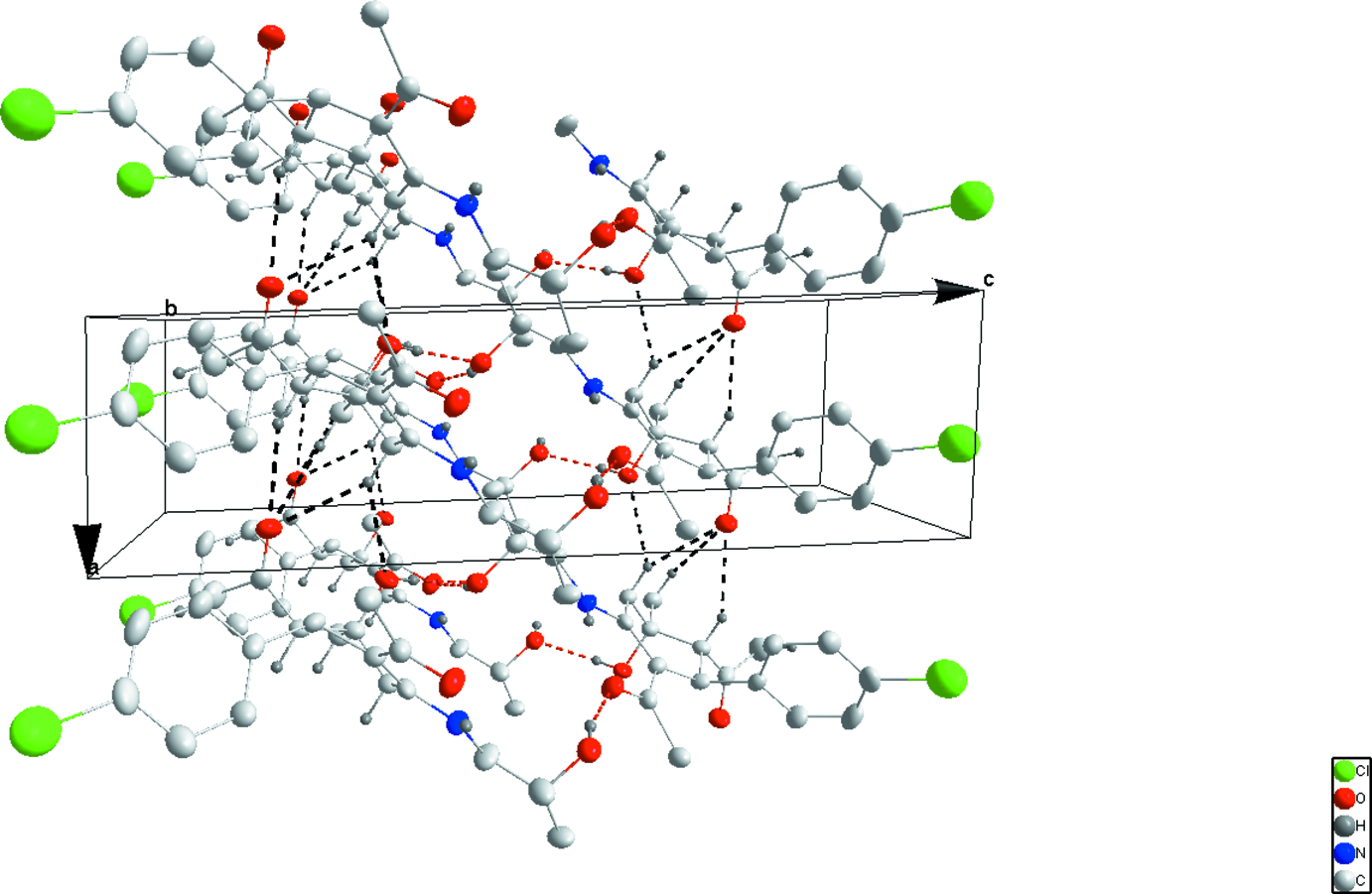

Supplement: Supplementary file 6 [file e-71-0o369-fig3.tif]
